# Preeclampsia and Gestational Hypertension: Biochemical and Antioxidant Features *in Vitro* Might Help Understand Different Outcomes

**DOI:** 10.1055/s-0041-1740270

**Published:** 2021-12-21

**Authors:** Victoria Elizabeth Galvão, Ricardo Carvalho Cavalli, Valeria Cristina Sandrim

**Affiliations:** 1 Department of Pharmacology, Instituto de Biociências, Universidade Estadual Paulista, Botucatu, SP, Brazil; 2 Department of Obstetrics and Gynecology, Faculdade de Medicina de Ribeirão Preto, Universidade de São Paulo, Ribeirão Preto, SP, Brazil; 3 Center for Toxicological Assistance, Instituto de Biociências, Universidade Estadual Paulista, Botucatu, SP, Brazil

**Keywords:** preeclampsia, potassium iodide, heme oxygenase-1, hypertension, endothelium, pré-eclâmpsia, iodeto de potássio, heme oxigenase-1, hipertensão, endotélio

## Abstract

**Objective** Gestational hypertension (GH) is characterized by increased blood pressure after the 20 ^th^ gestational week; the presence of proteinuria and/or signs of end-organ damage indicate preeclampsia (PE). Heme oxygenase-1 (HO-1) is an antioxidant enzyme with an important role in maintaining endothelial function, and induction of HO-1 by certain molecules shows potential in attenuating the condition's effects over endothelial tissue. HO-1 production can also be stimulated by potassium iodide (KI). Therefore, we evaluated the effects of KI over HO-1 expression in human umbilical vein endothelial cells (HUVECs) incubated with plasma from women diagnosed with GH or PE.

**Methods** Human umbilical vein endothelial cells were incubated with a pool of plasma of healthy pregnant women ( *n* = 12), pregnant women diagnosed with GH ( *n* = 10) or preeclamptic women ( *n* = 11) with or without the addition of KI for 24 hours to evaluate its effect on this enzyme expression. Analysis of variance was performed followed by Dunnet's test for multiple comparisons between groups only or between groups with addition of KI ( *p* ≤ 0.05).

**Results** KI solution (1,000 µM) reduced HO-1 in the gestational hypertension group ( *p* = 0.0018) and cytotoxicity in the preeclamptic group ( *p* = 0.0143); treatment with KI reduced plasma cytotoxicity but did not affect the preeclamptic group's HO-1 expression.

**Conclusion** Our findings suggest that KI alleviates oxidative stress leading to decreased HO-1 expression; plasma from preeclamptic women did not induce the enzyme's expression in HUVECs, and we hypothesize that this is possibly due to inhibitory post-transcriptional mechanisms in response to overexpression of this enzyme during early pregnancy.

## Resumo

**Objetivo** A hipertensão gestacional (GH) é caracterizada pelo aumento da pressão sanguínea após a 20ª semana de gestação; a presença de proteinuria e/ou sinais de danos a órgãos como rins, fígado e cérebro indicam pré-eclâmpsia (PE). A heme oxigenase-1 (HO-1) é uma enzima antioxidante com um papel importante na manutenção da função endotelial, e a sua indução por certas moléculas se mostra potencialmente benéfica frente à característica deletéria destas condições sobre o endotélio. Já foi demonstrado anteriormente que a produção de HO-1 pode ser induzida por iodeto de potássio (KI). Portanto, nós avaliamos os efeitos do KI sobre a citotoxicidade e expressão de HO-1 por células de veia de cordão umbilical humano (HUVECs) após incubação com o plasma de mulheres diagnosticadas com GH ou PE.

**Métodos** Células de veia de cordão umbilical humano foram incubadas com *pool* de plasma de gestantes saudáveis ( *n* = 12), gestantes com GH ( *n* = 10) ou gestantes com PE ( *n* = 11) com ou sem a adição de KI por 24 horas para avaliar a citotoxicidade através da dosagem de lactato desidrogenase e produção de HO-1 por ELISA. Foi realizada ANOVA seguida de teste de Dunnet para múltiplas comparações entre os grupos estudados, considerando significativos valores de *p* ≤ 0,05.

**Resultados** A solução de KI (1.000 µM) reduziu a produção de HO-1 no grupo GH ( *p* = 0.0018) e a citotoxicidade no grupo PE ( *p* = 0.0143); o tratamento com KI não afetou a produção de HO-1 por HUVECs incubadas com o plasma do grupo PE.

**Conclusão** Nossos achados sugerem que o KI atenua os efeitos do plasma de gestantes com GH ocasionando a diminuição da produção de HO-1; plasma do grupo PE não induziu a produção de HO-1 em HUVECs em comparação ao grupo saudável, e nossa hipótese é a de que tal achado pode ser devido a mecanismos pós-transcricionais em resposta a uma superestimulação da produção de HO-1 nos estágios iniciais da gravidez.

## Introduction

Gestational hypertension (GH) is characterized by high blood pressure after the 20th gestational week. 1 Around 25% of women diagnosed with GH on average will eventually develop preeclampsia, presenting additional hematological abnormalities and signs of end-organ damage such as edema, headaches, eyesight changes, difficulty breathing, and nausea. 1 2 Preeclampsia (PE) is the major cause of maternal death in Latin America and the Caribbean region with a worldwide incidence of 5 to 10% of all pregnancies. 3 4 5

These disorders' exact causes are still unknown; risk factors include obesity, nulliparity, and a family history for these conditions. 6 The most accepted hypothesis for the pathophysiology of preeclampsia consists of the “two-stage model,” in which it was proposed that an inadequate and inefficient placentation process early on in pregnancy leads to an ischemic, defective organ that eventually secretes vasoactive molecules affecting the endothelium, leading to the characteristic clinical signs and symptoms. 7 When GH does not progress to PE, it usually displays an intermediate phenotype between normal pregnancy and PE, suggesting a more benign origin and presenting as less of a threat for both mother and baby. 8

Prooxidant, vasoconstricting, and antiangiogenic factors are upregulated in this condition. 9 The endothelium responds by increasing the expression of antioxidant enzymes such as heme oxygenase-1 (HO-1), an inducible enzyme expressed in many tissues that converts the heme group into carbon monoxide, bilirubin, and free iron, molecules with direct or indirect antioxidant and vasoactive functions. 10 11 Heme oxygenase-1 has been implicated in the pathogenesis of several diseases, including pregnancy-induced hypertension, and the induction of the enzyme has been shown to improve markers for these conditions both in vitro and in an in vivo model for PE. 12 13 14

Beyond the well-known role in thyroid hormones production, potassium iodide is crucial in pregnancy and also exhibits antioxidant and antiinflammatory properties; 14 besides, it has been shown to be capable of increasing HO-1 expression in skin explants and cells exposed to ultraviolet rays. 15

Thus, the present study aimed to evaluate the effects of plasma from women affected by GE and PE over human umbilical vein endothelial cells (HUVECs) as well as the effects of potassium iodide (KI) regarding cytotoxicity, antioxidant capacity, and HO-1 expression.

## Methods

### Source of Biological Samples

In the present study, we used plasma samples from a previous work aiming to compare clinical and laboratory characteristics, obstetric, and perinatal outcomes of patients with PE or GH. 16 A group of pregnant women were recruited at the ambulatory clinic of the University Hospital of the Faculdade de Medicina de Ribeirão Preto. This previous study was approved by the Institutional Review Board at Ribeirão Preto Medical School, Brazil (reference 4682/2006, approved date June 20 ^th^ , 2006) and was performed following the principles of the Declaration of Helsinki. All subjects gave written informed consent.

To avoid misdiagnosis of GH and PE, the 419 patients enrolled were diagnosed retrospectively with PE and GH. The diagnosis criteria were used according to the American College of Obstetricians and Gynecologists, and they were: systolic blood pressure above 140 mmHg and diastolic blood pressure above 90 mmHg on two different occasions at least 4 hours apart or systolic and diastolic blood pressure of 160 mmHg and 110 mmHg respectively after the 20 ^th^ gestational week in women with previously normal blood pressure readings, plus proteinuria; in the absence of the latter, newly onset hypertension accompanied by symptoms and laboratory findings such as neurological and visual impairment, pulmonary edema, thrombocytopenia, and impaired liver and renal function were used to establish a diagnosis. 1 High blood pressure (systolic ≥ 140 mmHg; diastolic ≥ 90 mmHg) after the 20 ^th^ gestational week without proteinuria and/or the aforementioned symptoms and signs was diagnosed as GH. The exclusion criteria were twin pregnancy, hemostatic abnormalities, chronic hypertension, diabetes mellitus, fetal abnormalities, cancer, and cardiovascular, autoimmune, renal, and hepatic diseases. All blood samples were obtained after the 30 ^th^ gestational week. Venous maternal blood samples were collected in tubes containing heparin, which were then centrifuged (1,000 g for 3 minutes). Plasma was collected, sampled in 1,000 μL tubes, and stored at −80°C prior to use. Due to the limited plasma aliquots stocked and the small quantity needed to prepare the pool, we stipulated a minimum of 10 and a maximum of 15 samples per group.

### Preparation of KI Solution and Redox Titration

Potassium iodide was purchased from Sigma-Aldrich Brazil (Catalogue number 221945–100G – Sigma-Aldrich Brazil Ltda., Bauru, SP, Brazil). A KI solution was prepared by solubilizing it in deionized water and for the redox titration; we used sodium thiosulfate, previously titrated with a potassium iodate solution as a primary standard. The stock solution concentration was 58 mM. For the experiments, we diluted that solution in growth culture to achieve a final concentration of 100 µM and 1,000 µM.

### Human Umbilical Vein Endothelial Cell Culture

Human umbilical vein endothelial cells (ATCC, Virginia, USA; CRL-2873) were used for this in vitro model of hypertensive disorders of pregnancy. Cells were cultivated in sterile 25 cm ^2^ flasks using growth medium (Gibco, CA, USA) supplemented with fetal bovine serum 10% v/v (Gibco), 50 μg/ml penicillin, 50 μg/ml streptomycin, and 0.5 μg/ml amphotericin B (Gibco). For the experiments described ahead, cells were detached from culture flask (Corning, Costar, Netherlands) using trypsin solution (Trypsin/EDTA 0.5/0.2 mg/ml in phosphate-buffered saline, PBS) centrifuged at 1,200 rpm for 10 minutes, resuspended in growth medium free from fetal bovine serum containing antibiotic and antifungal solution and seeded on 96 well microplate (1.10 4 cells/well) overnight at 37°C, 5% CO _2_ tension, and 95% humidity to ensure cell adhesion _._

### Incubation with Patients' Samples Pools and KI

Plasma samples from the healthy pregnancy (HP, *n* = 12), GH ( *n* = 10), and PE ( *n* = 11) groups were pooled by mixing equal volumes of each patient's plasma in 3 distinct 1,500 μL tubes (30 μL/patient). From the resulting volume, 240 μL of each pool was diluted in growth medium free from fetal bovine serum (160 μL growth medium/well) using three Falcon 15 mL centrifuge tubes (Sigma-Aldrich, St. Louis, MO, USA). Cell culture supernatant was discarded, and 180 μL of the diluted pools were added to each well to achieve a 10% pool of plasma concentration per well. Then, 20 μL of KI solution was added to the wells, resulting in the final concentrations of 10 µM, 50 µM, 100 µM, 500 µM, and 1,000µM, so the final volume would be 200 μL per well. Only the 100 µM and 1,000 µM final concentrations were selected due to their statistically relevant results. Cells incubated with growth medium and pool of plasma only were used as controls for each group, in which case the KI solution was not added, and the final volume was achieved by adding 20 μL of growth medium instead. After a 24-hour incubation period, the supernatant was collected for posterior cytotoxicity, total antioxidant capacity, and hHO-1 quantification assays.

### Cytotoxicity Assay

Cytotoxicity was assessed by measuring the lactate dehydrogenase (LDH) activity using the Pierce LDH cytotoxicity assay kit — catalogue number 88954 (Thermo Fisher Scientific, Waltham, MA, USA). Lactate dehydrogenase can be detected on cell culture supernatant when cell membrane integrity is lost indicating cytotoxicity; after a 24-hour incubation period with patients' pool, 30 µL of supernatant were collected and immediately transferred to another 96 well microplate, to which 30 µL of the substrate mix was added. After 30 minutes of incubation at room temperature while protected from light, 30 µL of stop solution was added, and the absorbance was read using 490 nm wavelength, with an additional 680 nm reading for background signal elimination.

### Total Antioxidant Capacity (TAC) and HO-1 Quantification

Total antioxidant capacity of culture supernatant was assessed using the Ferric Reducing Antioxidant Power (FRAP) assay. 17 The FRAP reagent was prepared by mixing 50 mL of 23 mM acetate buffer (pH = 3.6), 5 mL of 10 mM tripyridyltriazine (TPTZ) solution, and 5 mL 20 mM FeCl _3_ .6H _2_ O. A total of 10µL of the supernatant sample was added to a 96-well microplate, to which 290 µL of FRAP reagent were mixed, and the microplate was incubated at 37°C for 4 minutes. Absorbance was read at a 593 nm wavelength. The standard solution was prepared using Fe _2_ SO _4_ .7H _2_ O, and the unit used was mM equivalent Fe ^2+^ . The HO-1 quantification assay Human Total HO-1/HMOX1 DuoSet IC ELISA kit–catalogue number DYC3776 (R&D Systems, Inc., Minneapolis, MN, USA) was , and the experiment followed manufacturer instructions. The 450 nm wavelength was used for readings.

### Transmission Electron Microscopy (TEM)

Cells were seeded in tissue culture 6 wells plate (500,000 cells/well) overnight and incubated with HP, GH, and PE pool of plasma and with PE pool of plasma plus 1,000 µM of KI for 24 hours. Cells were removed from the trypsin-EDTA solution for a minute; the enzyme was neutralized with fetal bovine serum, and the cells were transferred to microtubes for centrifugation (1,200 rpm, 10 minutes). The supernatant was then discarded, and the cell pellet was suspended in Karnovsky fixative for another centrifugation. The process was repeated one more time before the cells were resuspended in more fixative and delivered at the Electron Microscopy Center of Universidade Estadual Paulista, Botucatu campus, for further processing.

### Statistical Analysis

The software used was GraphPad Prism 6 (GraphPad Software, San Diego, CA, USA). For statistical analysis of the clinical features of the patients enrolled, we used analysis of variance (ANOVA) followed by the Dunnet test for multiple comparisons when variables obeyed normal distribution or Kruskal-Wallis followed by the Dunn test for multiple comparisons when at least one group followed a non-parametric distribution. *P* -values are described in Table 1 .

**Table 1:** Clinical characteristics of pregnant women and delivery conditions

Parameters	HP	GH	PE	*p* -value
*N*	12	10	11	
Age (years)	22.3 (2.27)	21.3 (2.83)	21.6 (4.14)	ns
GW sampling	37.3 (1.33)	38.7 (1.94)	31.5 (3.62)*	* *p* < 0.0001
BMI sampling (kg/m ^2^ )	28.3 (2.28)	37.4 (7.78)*	28.9 (4.53)	* *p* = 0.0006
SBP sampling (mmHg)	113.8 (10.69)	126 (10.50)*	136.2 (16.14)**	* *p* = 0.0310; ** *p* = 0.0011
DBP sampling (mmHg)	71.83 (8.96)	80.40 (9.96)	90 (10.00)*	* *p* = 0.0001
Methyldopa (%)	0	100	90 (10/11)	–
Nulliparous (%)	41.6 (5/12)	100 (10/10)	100 (11/11)	–
GW delivery	39.5 (1.43)	40 (1.00)	34.5 (4.33)*	* *p* = 0.0005
Placental weight (g)	536.8 (106.28)	613 (146.29)	408 (160.88)	ns

Abbreviations: BMI, body mass index; DBP, diastolic blood pressure; GH, gestational hypertension; GW, gestational weeks; HP, healthy pregnant; PE, preeclamptic; SBP, systolic blood pressure.

*p* values in comparison with the HP group.

Data are shown as mean ± (SD) or median (underlined) ± (IQR).

Cell culture results were analyzed using ANOVA followed by the Dunnet test for multiple comparisons, with statistically significant *p* -values < 0.05. We compared the effects of plasma pool only over HUVECs in the three different groups as well as the effect of adding KI to culture using each group's pool of plasma.

### Ethical Approval and Consent of Participants

The present study was approved by the Institutional Review Board of the HCFMRP-USP (reference 4682/2006, approved date June 20, 2006). All participants provided written informed consent.

## Results

The clinical features of the patients from which plasma samples were obtained are shown in Table 1 . A total of 41.6% of healthy pregnant patients and all patients from the other two studied groups had no children prior to the study. Non-parametric distribution was found when analyzing systolic and diastolic blood pressure. No statistical differences were found between groups regarding age or placental weight; blood draws from preeclamptic patients were performed significantly earlier than the other two groups, and the group's average gestational week at delivery was significantly lower. Body mass index (BMI) was significantly higher in the GH group only. Systolic blood pressure was higher in both GH and PE groups, while diastolic blood pressure was significantly higher in the PE group only.

**Fig. 1:**
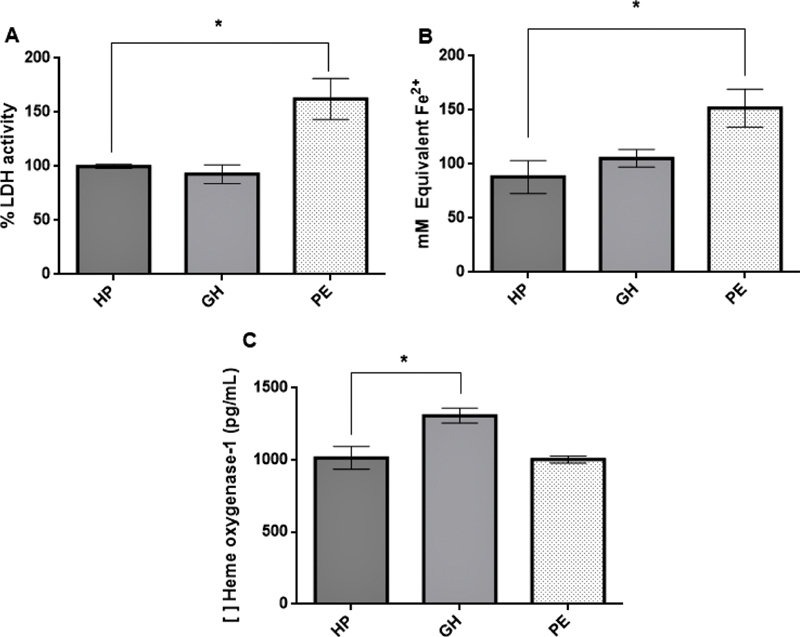
Cytotoxicity ( **A** ), total antioxidant capacity ( **B** ), and heme oxygenase-1 dosage ( **C** ) results. Human umbilical vein endothelial cells were incubated with the three studied groups' *pool* of plasma only. Healthy pregnant (HP) group's results were considered baseline values and were used as the control group for statistical analysis. Results are shown as mean ± SEM of quadruplicate wells per assay. (*) *p* < 0.05. The preeclamptic group's cytotoxicity and total antioxidant capacity results were significantly higher but showed the same capability of inducing heme oxygenase-1's expression. Gestational hypertension pool induced increased enzyme expression significantly.

The GH group did not show higher lactate-dehydrogenase activity compared with the healthy pregnant group. However, plasma from the PE group was significantly more cytotoxic ( *p* = 0.0227, Fig. 1A ). When treated with the highest KI concentration, cytotoxicity in the PE group was significantly reduced when compared with values from cell culture with PE plasma pool only ( *p* = 0.0143, Fig. 3A ).

**Fig. 2:**
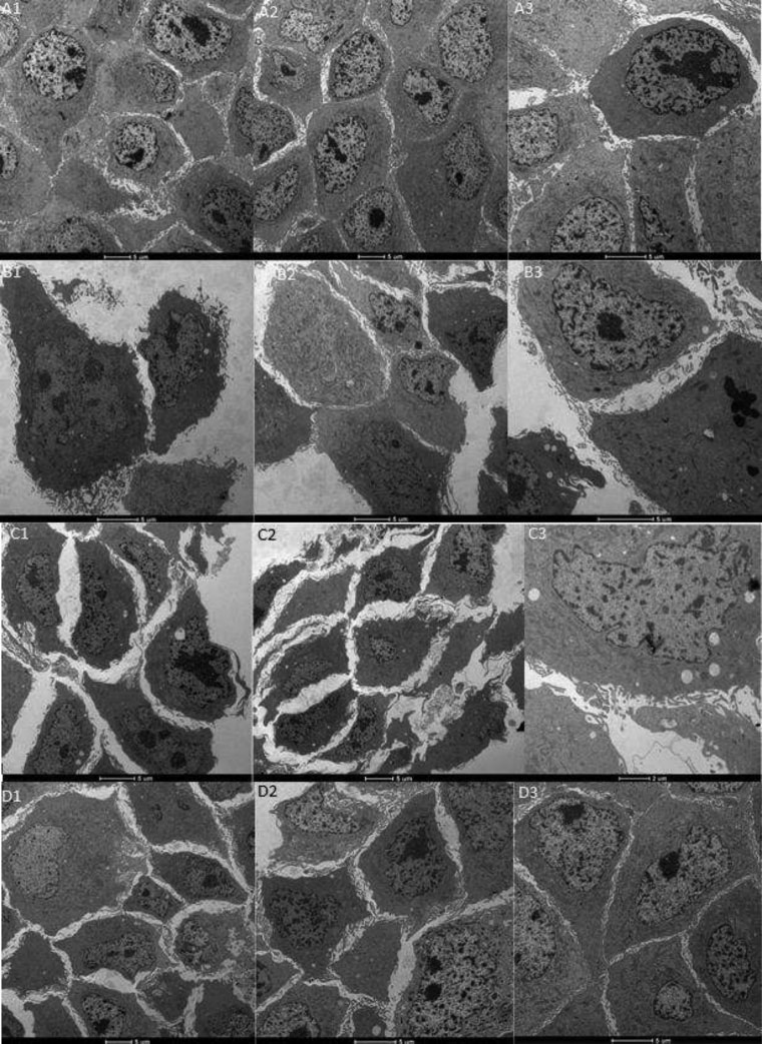
Transmission electron microscopy images of endothelial cells incubated with pool of plasma from healthy pregnant women (A1-A3), women with gestational hypertension (B1-B3), and preeclamptic women (C1-C3). Magnification of 5µm, except for C3 (2 µm). D1 to D3 show cells incubated with preeclamptic plasma and 1,000 µM KI. Images are representative of one assay and were captured throughout the entirety of the microscope field.

**Fig. 3:**
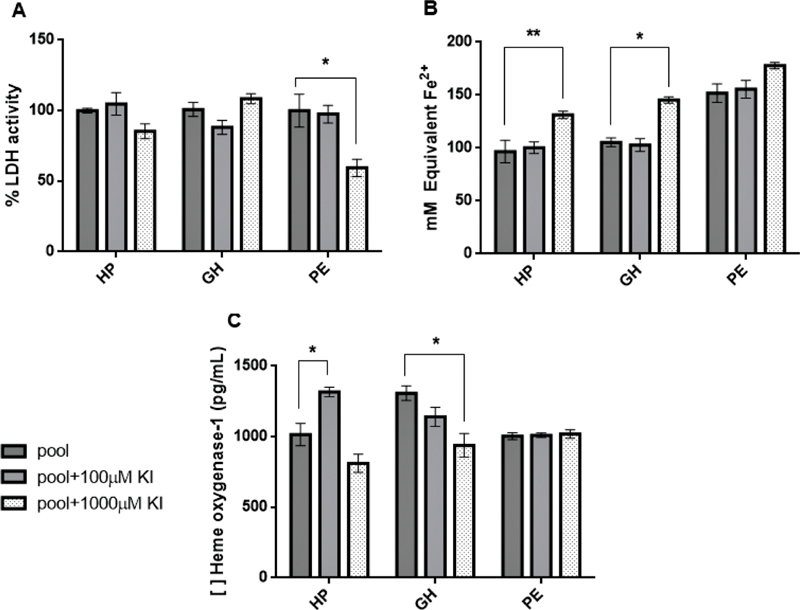
Cytotoxicity ( **A** ), total antioxidant capacity ( **B** ), and heme oxygenase-1 dosage ( **C** ) results. Human umbilical vein endothelial cells were incubated with the three studied groups' pool of plasma only (“pool *”* , used as control of each group) or pool of plasma from each group plus potassium iodide in two different concentrations. Results are shown as mean ± standard error of the mean (SEM) of quadruplicate wells per assay. (*) *p* < 0.01. (**) *p* < 0.005. Treatment with 1,000 µM KI reduced significantly the preeclamptic group's cytotoxicity and increased total antioxidant capacity in the healthy pregnant and gestational hypertension groups. Potassium iodide induced heme oxygenase-1's expression in healthy pregnant but reduced it significantly in gestational hypertension; the preeclamptic group's enzyme expression hasn't been affected by treatment.

Given the cytotoxicity results, we analyzed the cellular ultrastructure. Cells incubated with PE plasma plus 1,000 μM KI had different visual aspects compared with cells incubated with GH or PE plasma only ( Fig. 2 ). There is mild vacuolization in the cytoplasm in these groups, but the overall images shown in D1 to D3 resemble the healthy pregnant group images supporting our cytotoxicity results.

Samples from cells incubated with PE plasma only showed higher antioxidant capacity values when compared with the HP group ( *p* = 0.0021, Fig. 1B ). Supernatant from the HP and GH groups had increased antioxidant capacity when 1,000 µM KI solution was used compared with untreated groups ( *p* = 0.0006; *p* = 0.0025 respectively, Fig. 3B ). The antioxidant capacity of cells incubated with PE plasma plus KI did not change, even though there seems to be a tendency to its increase.

The results of HO-1 dosages from the GH group ( Fig. 1C ) showed increased expression of the enzyme compared with the HP group ( *p* = 0.074). Treatment with 100 µM KI induced HO-1 expression in the HP group ( *p* = 0.0065). The 1,000 µM treatment reduced the enzyme expression of the GH group when compared with results from cells incubated with this group's plasma pool only ( *p* = 0.0018, Fig. 3C ), but the preeclamptic group's HO-1 expression was not affected whatsoever by any concentration used.

## Discussion

In the present work, we found that GH plasma did not alter lactate-dehydrogenase release or antioxidant capacity of endothelial cells and that it induced HO-1 expression, while the opposite is seen in PE regarding the same parameters. It is remarkable, though, that KI reduced enzyme expression in the GH group, as well as lactate-dehydrogenase release in the PE group. Contrary to expected, HO-1 levels in the latter group were the same as in the healthy group, while the GH group showed increased levels, as an evidence of a preserved antioxidant mechanism induced by GH patients' plasma, which might explain the milder clinical features seen in Table 1 . In the PE group, this mechanism seems to be absent, even with increased total antioxidant capacity results. Iodine as an antioxidant, probably in a direct way by neutralizing oxidative species, as the treatment either reduced HO-1 expression or did not affect it whatsoever.

Both the GH and PE groups were already on methyldopa therapy at the time of sampling, which might explain the lack of statistical difference between the GH and HP groups' diastolic blood pressure values. Antihypertensive therapy offered no benefit to women from the PE group as both systolic and diastolic blood pressure measurements were significantly higher than the one from the HP group. Indeed, lack of responsiveness to antihypertensive therapy in PE women is associated with worse outcomes, and there have been studies addressing this observation using pharmacodynamics tools to further understand which personalized approach could be beneficial for these patients. 17 18

A previous study analyzed the oxidative stress markers and nitric oxide availability in the plasma of PE women and found a higher total antioxidant capacity corroborating with what was found in this work, as well as lower carbonyl levels, a biomarker for protein oxidation. Lipid peroxidation measured using the thiobarbituric acid reactive substances (TBARS) method was not different between groups, but it was lower in the PE group when compared with the GH group, with nitrite levels significantly lower in both hypertensive groups. 19 However, another study showed increased levels of 8-isoprostane and augmented oxidant stress in plasma from PE patients, but also found increased total antioxidant capacity, with no difference in vitamin E levels. 20 A meta-analysis published in 2018 found increased plasma oxidative stress markers in PE women, even though some studies used in this analysis showed an increase in plasma catalase and glutathione peroxidase, 21 suggesting that, in preeclampsia, the endogenous antioxidant defenses are indeed upregulated in later pregnancy stages, but this compensatory mechanism does not seem to be enough to counterbalance the systemic endothelial dysfunction caused by the disease. These observations support our results regarding lactate dehydrogenase release, as an increased antioxidant capacity in the preeclamptic group was not followed by lower cytotoxicity results.

Lactate dehydrogenase activity was increased, compared with the HP group, when cells were incubated using PE plasma only, with similar HO-1 results, while the GH group showed elevated HO-1 levels compared with the healthy group but with similar cytotoxicity results. This shows the cytoprotective effect of HO-1 in preserving cellular membrane integrity. Potassium iodide reduced lactate dehydrogenase activity in the PE and GH groups expression of HO-1, which shows that plasma from the latter group induces the enzyme expression and, therefore, protects endothelial cells, but with the addition of KI, endothelial cells responded by not increasing the enzyme expression. This effect might be due to the role of I ^-^ as a competitive substrate for oxidant enzymes such as myeloperoxidase, which were found to be elevated in cardiovascular disease as well as PE. 22 23 I ^-^ can also react with reactive oxygen species (ROS) such as hydrogen peroxide forming water and molecular iodine (I _2_ ), the latter being a stronger antioxidant than KI. 14 15 It has also been shown that peroxidases display catalytic activity in the presence of iodide, turning them into effective catalases, thus reducing peroxides in sera in vitro. 24 Further research is needed to assess which direct and indirect actions KI exerts to protect cell membranes from damage and, therefore, reduces cytotoxicity in vitro.

Our transmission electron microscopy images of endothelial cells showed slightly altered patterns in morphological structures of diseased groups, with a noticeable increase in cytoplasmatic vacuoles in the preeclamptic group; Focaccetti et al. 25 demonstrated that endothelial cells subjected to longer treatments with low doses of antineoplastic agent 5-Fluorouracil exhibited a pattern associated with cellular distress, similar to what was seen here. Preeclamptic plasma pool induced cells to a fusiform morphology and abundant membrane projections, which is mostly seen in distressed glioma cells 26 ; since the HP group showed a flat pattern with fewer cell projections, we may assume that what is seen in the PE group display an abnormal morphology, compared to healthy pregnant, which was reversed by KI.

It has been demonstrated that PE women have higher circulating HO-1. 10 27 However, incubation of HUVECs with PE plasma did not induce the enzyme's expression, differently from what was seen in GH women. Previous studies from our group found circulating levels of HO-1 to be increased in the plasma of pregnant women who later developed preeclampsia with severe features compared with women who developed preeclampsia without severe features; moreover, the plasma of pregnant women who later developed preeclampsia induced a 4-fold increase in the expression of HO-1 by HUVECs. 13 28 Another study from our group showed a similar pattern when assessing endothelin-1, a potent vasoconstrictor found elevated in PE women's plasma, as well as let-7 family micro ribonucleic acid (miRNA) targeting its transcripts. 29 Plasma endothelin-1 was found to be slightly increased before the onset of symptoms in the PE group and greatly increased after; circulating miRNA was increased in the PE group's plasma but not in the HP group's samples. The in vitro results showed that the plasma of women who later developed preeclampsia induced higher expression of endothelin-1 in HUVEC's before symptoms, but, after that, there was no difference between the HP and PE groups' endothelin-1 protein expression, followed by significantly increased miRNA targeting the protein's transcripts. 30 The authors concluded that protective posttranscriptional mechanisms were developed in PE women to prevent the expression of endothelin-1 in endothelial cells later on in pregnancy.

Despite the undeniable HO-1's role in homeostasis, there have been studies showing also the disadvantageous side of the chronic and/or overstimulation of this pathway in central nervous system diseases, as well as lung disease and metabolic syndrome. 31 32 33 34 It is possible that in PE women, the early and intense stimulation of HO-1 expression might also induce the development of posttranscriptional mechanisms to diminish its expression. Our data showed a better outcome in pregnant women with GH than in women with PE, which suggests that the severity of the condition might be associated with the loss of HO-1 induction over endothelial cells. Further research is needed to evaluate possible miRNA that could be involved in this effect, and if they are absent in HP and GH cases.

Iodine is imperative for fertility and fetal and child development. 35 36 In Brazil, mandatory iodization has improved the general iodine nutritional status and, yet, pregnant women are still at risk for iodine deficiency 37 38 ; at least one study showed excessive iodine in the urine and breast milk of pregnant and lactating Brazilian women. 39 This is, most likely, due to the excessive amount of table salt consumed in Brazil, on average, almost twice the recommended dose of 5 g/day, with almost 60% of the population consuming between 8 and 12 g/day. 40 The form of iodine added to salt in Brazil is potassium iodate, a much more stable form of iodine than KI, but it has been shown to increase lipid peroxidation in porcine thyroid follicles in doses ranging from 2.5 mM to 500 mM, while KI did not exhibit these effects until a dose of 50 mM was used 41 ; potassium iodate, then, might not play the same role as KI in preventing oxidative stress due to particular properties of this iodine form. Potassium iodide supplementation in iodine-deficient, overweight women led to a decrease in hypercholesterolemia, 42 and a 300 μg dose KI/day was shown to be harmless regarding thyroid hormones in euthyroid subjects and even exerted modest anti-inflammatory actions, 43 showing that KI might be better tolerated and helpful in hypertensive disorders of pregnancy, as these conditions share the common oxidative, proinflammatory features of cardiovascular disease, to which these women as predisposed later in life. 44 45

As of limitations found in the making of this research work, we highlight that the KI concentrations used here could not possibly reproduce circulating plasma concentrations as these were found to be around 30 μg/L; 46 hence, our results express potential mechanisms only in an experimental environment. We have not investigated whether the plasma of PE women contained known or novel miRNA targeting HO-1 transcripts; therefore, the aforementioned hypothesis was not tested and is still to be further analyzed. Likewise, we have not analyzed endothelial cells from either healthy or hypertensive pregnant women, which could also enlighten the differences in response to plasma molecules in the two studied groups.

## Conclusion

We conclude by highlighting the difference between GH and PE outcomes and effects over endothelial cells, an important distinction to be made when studying these conditions. Potassium iodide does have a protective effect over HUVECs when incubated with the plasma of both hypertensive groups that is independent of HO-1 activation, either by directly scavenging reactive species or by competing for oxidant enzymes as a substrate. Heme oxygenase-1 early overexpression in preeclampsia may play a detrimental role later on pregnancy; therefore, possible posttranscriptional mechanisms must be assessed to understand when this enzyme activation is beneficial in each condition studied. Further research is needed to evaluate if iodine can affect these parameters in vivo by reducing known biomarkers of oxidative stress and cytotoxicity.
